# Correction: Correlation between individual thigh muscle volume and grip strength in relation to sarcopenia with automated muscle segmentation

**DOI:** 10.1371/journal.pone.0327308

**Published:** 2025-06-30

**Authors:** Hyeon Su Kim, Shinjune Kim, Hyunbin Kim, Yonghan Cha, Jung-Taek Kim, Jin-Woo Kim, Yong-Chan Ha, Jun-Il Yoo

The Funding statement for this article is incorrect. The correct Funding statement is as follows: This research was supported by a grant of the Korea Health Technology R&D Project through the Korea Health Industry Development Institute (KHIDI), funded by the Ministry of Health & Welfare, Republic of Korea (grant number: HI22C0494).
