# Supplementary material for: Correlation between individual thigh muscle volume and grip strength in relation to sarcopenia with automated muscle segmentation
Source: PLoS One. 2024 Dec 26;19(12):e0312107. doi: 10.1371/journal.pone.0312107 (PMC11670947; doi:10.1371/journal.pone.0312107)
Supplement: Attachment — Submitted filename: PLOS_one_review_sarcopenia_aisegmentation.docx [file pone.0312107.s001.docx]

The article “Correlation Between Individual Thigh Muscle Volume and Grip Strength

In Relation to Sarcopenia: An AI-based Muscle Segmentation Using UNETR” adds the evidence of using AI-based segmentation of CT for value-added screenings. The authors show value by pushing limits of using AI in segmentation, especially one based on a transformer model, in clinical cases. This will help push the field and give an example of a segmentation algorithm that can assess and label multiple muscle groups. However, this reviewer has found multiple concerns that should be addressed before publication.

Minor concerns

- What are limitations of focusing only on grip strength as a functional outcome? EWGSOP does also mention other functional measures. Thigh volume correlation to grip strength is not as important, at least to this reviewer, as the relationship between thigh volume correlation and sarcopenia. Consider this as you write both the introduction and discussion.
- What about CT muscle density? Segmentation models exist to automatically evaluate muscle density, i.e. muscle quality, which has a higher association with sarcopenia than volume, i.e. muscle mass. This should be addressed as a potential limitation of the study as recommendations by EWGSOP prefer muscle quality over muscle mass.
- Change section title “Result” to “Results”
- Change “groped” to “grouped” for Fig 7 title
- The resolution of Fig 6 and 7 are low. A lot of information is packed into these figures, so when I zoom in to see trends and read labels, the font becomes blurry.
- Technical features of segmentation model. Did any cases fail or need to be run again? What is the run time for the analysis of your images? This can help advocate for the implementation of your model in a practical setting with real-world images.
- The discussion does not connect much to established literature.

Major concerns

- AI is not the main focus of this article or being tested. Consider changing the title as it makes the article seem more about using AI in a novel way instead of an existing segmentation model.
- Patients all have hip fractures, which is associated with sarcopenia. There may not be a true control group to test these associations fully. Would the trends still exist with patients without hip fractures? If so, these associations might be fully helpful in diagnosing sarcopenia.
- Not clear in the first paragraph of the study design if the UNETR model was previously trained in the cited article or trained using the current sample.
- In the results of the point biserial correlation, the p-values are important but do not show the strength of correlation. What is the correlation value between the muscle groups and sarcopenia? Please report the correlation values in addition to the p-values. Without these, I cannot assess the strength of relationships between sarcopenia and thigh muscles.
- There are no y-axis labels in Fig 6.
- In Fig 6 and Fig 7, change your titles and x-axis to human readable labels instead of variable names.
- Results section for quantiles of men and women define 3 quartiles instead of 4 as displayed in Fig 6 and 7.
- Fig 6 and 7 are unclear if adjusted or raw muscle volumes are being used for correlations. State this is in the results as well.
- In results, please note that the noteworthy trend was not test statistically.
- In results, note the lack of trends among women’s muscle volume and grip strength. This is important finding especially as EWGSOP is recommending muscle performance for sarcopenia diagnosis.
- In results, the sentence “These findings suggest a subtle yet discernible correlation between anterior muscle volume and grip strength.” Should be changed to “trend between…” as no correlation was tested.
- A lot of the issues of “trends” in Figure 6 and 7 could be tested by comparing the muscle group volumes and ratios to grip strength continuously. You would be able to assess trend strength instead observing possible trends. You could also represent these in tables, which would likely make your results more readable.
- The last paragraph of the results states there are differences and associations, but none have been tested statistically between grip strength and muscle volumes. This reviewer highly recommends changing this language to “trends” or testing them with Pearson’s correlations and/or ANOVA tests.
- Please clarify the second statement in the discussion. Is this a correlation between muscle volumes and grip strength or muscle volumes and sarcopenia? If this is reference to muscle volumes and grip strength, that association was not tested as far as this reviewer can tell.
- The statement in the discussion- “The quantile analysis further supported these findings showing a clear trend in the male group that higher muscle volumes are correlated with better grip strengths.”- is not supported in the research. No correlation was established statistically.
- This reviewer would also like to see the point biserial correlations and any of the muscle volume comparisons of the ground truth against the AI segmentation to see how the differences compare to a performance metric (grip strength) and diagnosis of sarcopenia. If this is included, this reviewer feels the title including AI would be more valid.
