# Supplementary material for: Correlation between individual thigh muscle volume and grip strength in relation to sarcopenia with automated muscle segmentation
Source: PLoS One. 2024 Dec 26;19(12):e0312107. doi: 10.1371/journal.pone.0312107 (PMC11670947; doi:10.1371/journal.pone.0312107)
Supplement: Attachment — Submitted filename: [Response] PLOS_One Review_240219.docx [file pone.0312107.s002.docx]

**Response:**

**Minor concerns**

1. ***What are limitations of focusing only on grip strength as a functional outcome? EWGSOP does also mention other functional measures. Thigh volume correlation to grip strength is not as important, at least to this reviewer, as the relationship between thigh volume correlation and sarcopenia. Consider this as you write both the introduction and discussion.***

**Response:**

The consensus of EWGSOP diagnoses and determines the severity of sarcopenia by measuring grip strength, chair stand test, SPPB and gait speed to assess physical performance. The current guideline or diagnosing sarcopenia involves initially measuring grip strength and conducting a chair stand test to identify a ‘sarcopenia probable’ status, which is then confirmed through skeletal muscle index from Dual-Energy X-ray Absorptiometry (DXA). However, in our current study, we only had access to grip strength measurements in physical performance measurements for hip fracture patients. The majority of these patients, upon hospital admission, were unable to walk or undergo tests to evaluate their physical performance (SPPB, chair stand test and gait speed). So, we utilized grip strength to determine a ‘sarcopenia probable’ status and employed DXA scanning for the definitive diagnosis of sarcopenia.

2. ***What about CT muscle density? Segmentation models exist to automatically evaluate muscle density, i.e. muscle quality, which has a higher association with sarcopenia than volume, i.e. muscle mass. This should be addressed as a potential limitation of the study as recommendations by EWGSOP prefer muscle quality over muscle mass.***

**Response:**

We recognize the importance of assessing adipose tissue, both intermuscular and intramuscular, in the elderly and its impact on their physical function. However, our approach to developing the model involved using the labeled dataset, created by a threshold for fat tissues as background class. This process leads to a model that identifies muscle tissue exclusively, neglecting any fat present within the muscle. This limitation leads us to challenges to precisely evaluate the percentage of muscle fat infiltration in the outputs from our segmentation model. Currently, our research is focused on the volume of anatomical individual muscles at each functional site.

3. ***Change section title “Result” to “Results”***

**Response:**

I appreciate your pin out. I have changed the section title of “Result” to “Results”

4. ***Change “groped” to “grouped” for Fig 7 title***

**Response:**

Thank you for the pointing out the typo. I modified the title of Figure 7 to “Grouped thigh muscle distribution in quantile grip strength”

5. ***The resolution of Fig 6 and 7 are low. A lot of information is packed into these figures, so when I zoom in to see trends and read labels, the font becomes blurry.***

**Response:**

Thank you for your feedback. I have noticed that Figure 6 and 7 become blurry when I zoom in. I have uploaded the Figure 6 and 7 with higher resolution.

6. ***Technical features of segmentation model. Did any cases fail or need to be run again? What is the run time for the analysis of your images? This can help advocate for the implementation of your model in a practical setting with real-world images.***

**Response:**

I appreciate your concern. Basically, some cases include any implants, amputation or fracture of femoral shaft which make high artifact on imaging or anatomical failure can occur low accuracy of segmentation. We excluded those cases from our criteria. To advocate for the implementation of the AI-based segmentation model, we provide two supplemental files, the figure of segmentation result image of specific slices of each patient and the running time of the segmentation process.

**Major concerns**

1. ***AI is not the main focus of this article or being tested. Consider changing the title as it makes the article seem more about using AI in a novel way instead of an existing segmentation model.***

**Response:**

Thank you for the feedback. As your recommendation, I changed the title to “Correlation Between Individual Thigh Muscle Volume and Grip Strength In Relation to Sarcopenia with Automated Muscle Segmentation”

2. ***Patients all have hip fractures, which is associated with sarcopenia. There may not be a true control group to test these associations fully. Would the trends still exist with patients without hip fractures? If so, these associations might be fully helpful in diagnosing sarcopenia.***

**Response:**

Thank you for your valuable feedback. The study utilized data from Real Hip Cohort of hip fracture patients. The analysis was based on CT scans, clinical findings and examinations obtained from these hip fracture patients. Unfortunately, we were unable to acquire CT scans from the general population under the same settings. However, in response to your insightful comment, we plan to include data from the general population by funding for future research projects. Regarding this limitation mentioned, we will incorporate the following statement in the Limitations section.

“Secondly, the study primarily focused on patients with hip fractures, a group inherently linked to sarcopenia, hence lacking a comparative control group without hip fractures to fully evaluate these associations. This limitation, along with an imbalance in our dataset concerning sarcopenia diagnosis because hip fractures are more common in older individuals who are also more likely to have sarcopenia, could potentially lead to biased results[33–35].

Further research is warranted to determine whether the observed trends would persist in individuals without hip fractures, as such findings could potentially enhance the utility of these measures in diagnosing sarcopenia. In future studies, it would be useful to examine if the observed trends continue in individuals without hip fractures, as this could help refine the application of these metrics in diagnosing sarcopenia.”

3. ***Not clear in the first paragraph of the study design if the UNETR model was previously trained in the cited article or trained using the current sample.***

**Response:**

Thank you for your valuable feedback. For any confusion, we modified the sentences.

**“By employing the advanced deep learning framework UNETR, tailored for meticulous voxel-level segmentation and sequential data interpretation, we trained the model to achieve notable metrics with 72 CT scan datasets in our previous study. Specifically, our UNETR model attained a Dice score of 0.84 and a relative average volume difference of 0.019%”**

4. ***In the results of the point biserial correlation, the p-values are important but do not show the strength of correlation. What is the correlation value between the muscle groups and sarcopenia? Please report the correlation values in addition to the p-values. Without these, I cannot assess the strength of relationships between sarcopenia and thigh muscles.***

**Response:**

Thank you for the point out of the strength of correlation. We provided strength of correlation in the Table 1, which is not proper expression like correlation. We changed it as Correlation coefficient. Thank you.

5. ***There are no y-axis labels in Fig 6.***

**Response:**

Thank you for your feedback. I have revised Figure 6 to make the y-axis.

6. ***In Fig 6 and Fig 7, change your titles and x-axis to human readable labels instead of variable names.***

**Response:**

Thank you for the feedback. I’ve updated the titles and the x-axis in Figure 6 and 7 to display more intuitive labels.

7. ***Results section for quantiles of men and women define 3 quartiles instead of 4 as displayed in Fig 6 and 7.***

**Response:**

Thank you for your insightful feedback. Upon reflection there might be confusion regarding how the quantiles were presented in the Results section. To address this, I have revised the text to all four quantiles clearly.

“The first quantile (Q1) was at 18kg, the median (Q2) stood at 20kg, the third quantile (Q3) was at 26.3kg and the fourth quantile (Q4) was 37kg. In contrast, the female cohort had a mean grip strength of 12kg, accompanied by a standard deviation of 5.14kg. The first quantile (Q1) was at 5kg, the median (Q2) was at 11.6kg, the third quantile (Q3) was 14.72kg and the fourth quantile (Q4) was 27.6kg.”

8. ***Fig 6 and 7 are unclear if adjusted or raw muscle volumes are being used for correlations. State this is in the results as well.***

**Response:**

**Thank you for your feedback. To set the linearity of skeletal muscle index, We utilized adjusted volume data for Figure 6 and 7. I have clarified the information in the Results section.**

**“Subsequent to adjusting for muscle volumes by height (volume/height^2^), as illustrated in Figure 6, a trend was observed exclusively within the male group, where greater muscle volumes were associated with higher grip strengths.”**

9. ***In results, note the lack of trends among women’s muscle volume and grip strength. This is important finding especially as EWGSOP is recommending muscle performance for sarcopenia diagnosis.***

**Response:**

Thank you for your valuable feedback. As your suggestion, we emphasize the result of the lack of trends among women’s muscle volume and grip strength. The following paragraph is the modified paragraph in the “Results” section.

“As illustrated in Figure 6, a noteworthy trend was observed exclusively within the male group, where greater muscle volumes were associated with higher grip strengths. Furthermore, as shown in Figure 7, the anterior muscle group displayed specific mean volumes across different quartiles: 68.95 in the second quartile (Q2), 93.83 in the third quartile (Q3), and 107.02 in the fourth quartile (Q4). These findings suggest a subtle yet discernible correlation trend between anterior muscle volume and grip strength in male group. This pattern was not observed in the female group, where no significant relationship with muscle volume was found (p > 0.05).”

10. ***In results, the sentence “These findings suggest a subtle yet discernible correlation between anterior muscle volume and grip strength.” Should be changed to “trend between…” as no correlation was tested.***

**Response:**

Thank you for your correction of the sentence in “Results” section. We modified the sentence like the following sentence.

“

These findings suggest a subtle yet discernible trend between anterior muscle volume and grip strength.

”

11. ***A lot of the issues of “trends” in Figure 6 and 7 could be tested by comparing the muscle group volumes and ratios to grip strength continuously. You would be able to assess trend strength instead observing possible trends. You could also represent these in tables, which would likely make your results more readable.***

**Response:**

Thank you for your valuable suggestions on enhancing the statistical presentation. We conducted a Spearman's rank correlation analysis to explore the relationship between grip strength and the volume of each specific thigh muscle. To improve readability and present our findings more effectively, we've incorporated these results into Table 3, titled "Spearman's Rank Correlation between Grip Strength and Individual Thigh Muscle Volume."

12. ***The last paragraph of the results states there are differences and associations, but none have been tested statistically between grip strength and muscle volumes. This reviewer highly recommends changing this language to “trends” or testing them with Pearson’s correlations and/or ANOVA tests.***

**Response:**

I appreciate your valuable input. I have modified the context of correlation to trends in the paragraphs within the Results section.

13. ***Please clarify the second statement in the discussion. Is this a correlation between muscle volumes and grip strength or muscle volumes and sarcopenia? If this is reference to muscle volumes and grip strength, that association was not tested as far as this reviewer can tell.***

**Response:**

Thank you for your insights. To avoid any confusion, I have made it clear that this is about the correlation between thigh muscle volumes and sarcopenia, according to the current consensus. Thank you for pointing this out.

“The present study employed grip strength and skeletal muscle index as a consensus marker for diagnosing sarcopenia and found significant correlations between sarcopenia and thigh muscle volumes such as Rectus femoris, Vastus lateralis and Vastus intermedius, particularly in the male cohort. Those specific thigh muscles imply their potential contribution in the onset or progression of sarcopenia in males. Conversely, no significant associations were observed between sarcopenia and each individual muscle volume in female group.”

14. ***The statement in the discussion- “The quantile analysis further supported these findings showing a clear trend in the male group that higher muscle volumes are correlated with better grip strengths.”- is not supported in the research. No correlation was established statistically.***

**Response:**

Thank you for your guidance. Following your suggestion, I’ve revised the sentence to reflect that we did not conduct a correlation analysis.

“

The analysis of quantile indicates a trend within the male group where increased volumes of the anterior and medial thigh muscles could be linked with stronger grip strength.

”

15. ***This reviewer would also like to see the point biserial correlations and any of the muscle volume comparisons of the ground truth against the AI segmentation to see how the differences compare to a performance metric (grip strength) and diagnosis of sarcopenia. If this is included, this reviewer feels the title including AI would be more valid.***

**Response:**

Providing an analysis with the ground truth would indeed be valuable. However, we employed a different dataset in Real Hip Cohort for model development which included 60 CT scans for training and 12 CT scans for validation. Creating a ground truth segmentation for a single CT scan requires approximately 4 hours, generating ground truths for all 49 scans would be exceedingly labor-intensive and costly. Consequently, it is difficult to compile a complete set of ground truth data for analysis. We apologize for our inability to present a full ground truth analysis under these conditions.

**Introduction:**

1. ***In the introduction, it would be beneficial to include a brief explanation of UNETR and why it is a more accurate choice for assessing individual thigh muscle volumes. This will help to avoid any potential confusion.***

**Response:**

Thank you for your feedback. I have included a brief explanation of UNETR and previous study to avoid any potential confusion.

“

The UNETR model represents an AI-driven semantic segmentation architecture that merges the U-net and Transformer modes. It excels in accurate voxel-level segmentation of 3D medical images, including CT scans and MRIs. In our previous study, we utilized the UNETR architecture to develop a thigh muscle segmentation model, employing a dataset of 72 entries (60 for training and 12 for validation) from the Real Hip Cohort. Real Hip Cohort includes variety of medical images, such as CT scans, X-rays and Dexa, along with physical performance metrics like grip strength from hip fracture patients.

”

2. ***The introduction lacks an explanation and reference for why the thigh muscles have a direct impact on grip strength (Ln 65-67). This oversight makes it difficult to understand the primary aim mentioned later (Ln 83-85), particularly the relationship between thigh muscle volumes, grip strength, and sarcopenia, as well as the reason for investigating these factors.***

**Response:**

Thank you for the feedback. We have updated the paragraph of relationship between thigh muscles and grip strength more logical.

“

Beyond the focus on the hand and forearm muscles in grip strength, other muscle groups like those in the thigh also play an essential role[10]. A loss of balance and stability could potentially compromise the ability to exert force effectively through one’s grip[11]. The gluteus muscles are integral for maintaining balance[12]. Drawing on the insights from these studies, balance-critical muscles, including the lower body and thigh muscles, may have a secondary effect on grip strength by promoting enhanced stability and strength. Based on the findings from these studies, it is hypothesized that lower body muscles, including those in the thigh, contribute to stability and power, which could have an indirect effect on grip strength.”

3. ***Ln 116-118, “we aim to provide a nuanced understanding of how individual thigh muscles, once adjusted for volume, may be implicated in the onset or progression of sarcopenia when diagnosed through grip strength criteria.” Including this statement in the introduction would provide the audience with a more comprehensive understanding of the study's intentions.***

**Response:**

To prevent future confusion, I have removed the term of ‘once adjusted for volume’,

“By employing the point-biserial correlation method, we aim to provide a nuanced understanding of how individual thigh muscles, once adjusted for volume, may be implicated in the onset or progression of sarcopenia when diagnosed through grip strength criteria.”

Additionally, I have incorporated a statement regarding how sarcopenia progresses in relation to the volumes of individual thigh muscles.

“The primary aim of this study is to investigate the relationship between individual thigh muscle volumes and grip strength in diagnosing sarcopenia. To achieve this, we employ a UNETR-based segmentation approach, enhancing the efficiency and precision in measuring the volumes of individual thigh muscles.”

4. ***Ln 124-127, “By examining this relationship, we seek to uncover patterns or trends that may offer valuable insights into how the proportional volumes of thigh muscles can influence grip 126 strength. This is particularly relevant in the context of sarcopenia, where both muscle mass and functional performance measures like grip strength are of critical importance” should also be mentioned concisely in introduction.***

**Response:**

Thank you for the feedback. We have updated the introduction mentioning the context.

“Acknowledging this complexity interplay between various thigh muscle groups and the evolving understanding of sarcopenia, the purpose of this study is to discover the correlation relationship between individual thigh muscle and grip strength. This insight is crucial for understanding sarcopenia, where muscle mass and functional metrics such as grip strength play key roles. This emphasize on muscle mass and function highlights the necessity of precise individual muscle size measurement in a functional context.”

5. ***In Line 54-57, the two sentences repeat the same message and are nearly identical in terms of writing. The authors should consider amending the second sentence to provide reasons for why sarcopenia can significantly impact the quality of life in older adults.***

**Response:**

Thank you for pointing out the repetitive content. I have removed the second sentence of “It is a syndrome characterized older adults.~.

6. ***In Line 62, the authors state that "EWGSOP2 and AWGS2019 incorporated grip strength as a consensus measure for diagnosing and assessing the severity of sarcopenia". This statement is inaccurate and is a critical concern of this manuscript. A key assumption of this study is that the association between thigh muscle and grip strength can inform the role of thigh muscle in sarcopenia due to grip strength being a consensus measure of sarcopenia. However, grip strength alone cannot be used to diagnose or assess the severity of sarcopenia. Indeed, the operational definition of sarcopenia according to the European Working Group on Sarcopenia (EWGSOP2) and the Asian Working Group for Sarcopenia (AWGS2019) requires low grip strength to be accompanied by either low muscle quantity or quality for EWGSOP2 and low appendicular skeletal muscle mass for AWGS2019. Please provide a detailed justification for why grip strength independently was used to diagnose sarcopenia in this study.***

**Response:**

Thank you for your insightful feedback. You are right, and I have amended the sentence you pointed out to reflect screening practices. “In 2019, both the European Working Group on Sarcopenia in Older People 2 (EWGSOP2) and the Asian Working Group for Sarcopenia (AWGS2019) incorporated grip strength as a consensus measure for screening of possible sarcopenia[8,9].”

Additionally, to diagnose sarcopenia, participants’ skeletal muscle index was measured for confirmation, a detail I have included in the Study Design section.

“Sarcopenia diagnosis in our study is based on grip strength (female<18kg and male<28kg) and skeletal muscle index using Dual-energy X-ray absorptiometry (female<5.4kg and male<7.0kg), following the guidelines set by Asian Working Group for Sarcopenia (AWGS), aligning with evolving consensus in the field that emphasizes functional performance measures alongside muscle mass[8].”

7. ***In Line 67-68, the authors state that "gluteus muscles are integral for maintaining balance, a factor that might also have an impact on grip strength". In fact, the referenced study does not include grip strength as an outcome nor mention grip strength in their article. The authors should justify this statement and amend the reference.***

**Response:**

Thank you for your insight. I have noticed now that the sentences may have overstated the result of referenced study. I have revised the paragraph.

“The gluteus muscles are integral for maintaining balance[12]. Drawing on the insights from these studies, balance-critical muscles, including the lower body and thigh muscles, may have a secondary effect on grip strength by promoting enhanced stability and strength.”

8. ***The authors have highlighted the limitations of previous methods to assess muscle size and promoted UNETR as a potential alternative. However, the authors did not explain what UNETR is, and why it might serve as a more valid and efficient method to assess muscle volume. Therefore, the rationale for using the UNETR technique is missing. As the research gap identified is to investigate the correlation of thigh muscle volume assessed via the UNETR technique with grip strength, it is crucial to identify the importance of using the UNETR technique.***

**Response:**

Thank you for your feedback. I have included a brief explanation of UNETR and previous study to avoid any potential confusion in the Introduction section.

“The UNETR model represents an AI-driven semantic segmentation architecture that merges the U-net and Transformer modes. It excels in accurate voxel-level segmentation of 3D medical images, including CT scans and MRIs. In our previous study, we utilized the UNETR architecture to develop a thigh muscle segmentation model, employing a dataset of 72 entries (60 for training and 12 for validation) from the Real Hip Cohort. Real Hip Cohort includes variety of medical images, such as CT scans, X-rays and Dexa, along with physical performance metrics like grip strength from hip fracture patients.”

**Methods:**

9. ***To ensure a comprehensive and unbiased account of the study's outcomes, it is essential to include and describe the findings of both the male and female cohorts in the results section. Even if the findings for the female cohort are nonsignificant, their inclusion provides a complete picture of the study's results and their potential implications for both genders.***

**Response:**

Thank you for your valuable insight. I have included the findings of female group in the Results section.

“Conversely, no significant correlations were observed between sarcopenia and any individual muscle in the female group.”

“This pattern was not observed in the female group, where no significant relationship with muscle volume was found (p > 0.05).”

10. ***In Line 94, the authors state that participants were from a "cohort of hip fracture patients". This is another critical concern, as hip fracture patients are likely to have experienced thigh muscle atrophy during their recovery period, and therefore have lower thigh muscle volume as a result of their injury. This can strongly bias the findings of the study. Please clarify and justify why participants were selected from a cohort of hip fracture patients.***

**Response:**

Thank you for highlighting this concern. It’s important to note that all CT scans analyzed in this study were pre-operative and all taken within 24 hours of the hip fracture occurrence. This timing minimizes the potential for muscle atrophy affecting our findings, as the scans were captured shortly after the injury, before any recovery period. To prevent any future confusion, I have added the information of the CT scan in the CT scans Acquisition subsection of the Methods section.

“In this study, the CT scans utilized were from participants underwent pre-operative CT scans, all conducted within 24 hours following the hip fracture incident.”

11. ***In Line 113, the authors state that the diagnosis of sarcopenia is based on grip strength. As mentioned previously, the diagnosis of sarcopenia according to the AWGS2019 definition requires that the individual have low appendicular skeletal muscle mass coupled with low muscle strength (i.e., low grip strength). The authors should consider reassessing the presence of sarcopenia in their study sample while complying fully with the AWGS2019 definition.***

**Response:**

Thank you for your insightful comment regarding the diagnostic consensus for sarcopenia. We have adhered to the consensus guidelines for diagnosing sarcopenia, which involve assessing the skeletal muscle index using Dexa. I inadvertently overlooked this detail in the manuscript, but I have now updated the paragraph describing the sarcopenia diagnosing process in the Introduction section.

“Sarcopenia diagnosis in our study is based on grip strength (female<18kg and male<28kg) and skeletal muscle index using Dual-energy X-ray absorptiometry (female<5.4kg and male<7.0kg), following the guidelines set by Asian Working Group for Sarcopenia (AWGS), aligning with evolving consensus in the field that emphasizes functional performance measures alongside muscle mass[8].”

12. ***In Line 134, the authors refer to Figure 1 for the inclusion criteria. Figure 1 is the flowchart of the study. The authors should include a table for eligibility, detailing the inclusion and exclusion criteria.***

**Response:**

Thank you for the feedback. I have added Table 1 for detailed criteria.

**Results:**

13. ***The authors should include the baseline demographic and characteristics of the participants.***

**Response:**

Thank you for the feedback. I have included the baseline demographic and characteristics of the participants.

“In this study, the participant cohort exclusively consisted of individuals with hip fractures, totaling 49 participants. Among them, 32 were females, while 17 were males. The average age of the female group was 77.3 years, with a standard deviation of 10.46, while the average age of the male group was 75.6 years, with a standard deviation of 6.42.”

14. ***Results for the correlation between sarcopenia (diagnosed using grip strength independently) and individual thigh muscles for the female group were not reported. This omission is misleading and may cause the reader to presume that thigh muscle volume is strongly correlated with sarcopenia when this finding only holds true in the male group. Indeed, upon reviewing Table 1, none of the individual thigh muscles was significantly correlated with sarcopenia in the female group. Please include the findings for the female group in the results section.***

**Response:**

Thank you for your input. I have now incorporated the results from the female group, which showed no significant correlation with any individual thigh muscle volume.

“Conversely, no significant correlations were observed between sarcopenia and any individual thigh muscle in the female group.”

**Discussion:**

15. ***The discussion section recommends targeted physical activity to maintain stable gait and overall physical agility in older populations. However, the study did not assess gait or physical agility, and therefore the recommendation lacks support. The discussion would benefit from including a reference to support the relationship between thigh muscle composition and gait/physical agility to strengthen the recommendation.***

**Response:**

Thank you for the valuable feedback. I have included references for the relationship between thigh muscle and gait/physical agility.

“Specifically, tailored exercises that target particular thigh muscle groups might offer more substantial benefits for maintaining stable gait and overall physical agility in older populations[23–25].”

References

[

23. Abe T, Ogawa M, Loenneke JP, Thiebaud RS, Loftin M, Mitsukawa N. Relationship between site-specific loss of thigh muscle and gait performance in women: The HIREGASAKI study. Archives of Gerontology and Geriatrics. 2012;55: e21–e25. doi:10.1016/j.archger.2012.06.009

24. de Abreu DCC, Cliquet A, Rondina JM, Cendes F. Electrical Stimulation During Gait Promotes Increase of Muscle Cross-sectional Area in Quadriplegics: A Preliminary Study. Clin Orthop Relat Res. 2009;467: 553–557. doi:10.1007/s11999-008-0496-9

25. Lindemann U, Mohr C, Machann J, Blatzonis K, Rapp K, Becker C. Association between Thigh Muscle Volume and Leg Muscle Power in Older Women. PLoS One. 2016;11: e0157885. doi:10.1371/journal.pone.0157885

]

16. ***The discussion section should further analyze and interpret the possible reasons for the non-significant result of the female cohort, such as sample size, hormonal differences, or other variables. This will provide valuable insights and contribute to the overall interpretation of the study's findings.***

**Response:**

Thank you for your insightful feedback on the potential gender-based differences. In response, I have included a paragraph in the Discussion section that further elucidates these gender-specific distinctions.

“In the result of correlation between thigh muscle volume and sarcopenia, the lack of a significant in female group may stem from the interplay of hormonal mechanisms across genders. Regarding hip fractures, the timing of peak bone and muscle mass loss, leading to increased fragility of hip fractures after this peak intersection point[26-29]. For men, the sequence typically involves a decrease in muscle mass due to lower testosterone levels before bone loss occurs[30]. In contrast, in women, the process of muscle loss generally occurs after the loss of bone density, which is influenced by estrogen levels[31,32]. This suggests that for women, muscle mass might not significantly influence the progression of sarcopenia in the context of hip fractures. However, this conclusion could be affected by a potential bias due to a limited sample size in the study group.”

17. ***Due to the small sample size and the predominance of findings in males, the study's generalizability is limited. Describing the relationships as "promising" may be misleading. It is important to consider these limitations and interpret the findings cautiously.***

**Response:**

I appreciate your valuable feedback. Based on your comments, I have revised the entire Conclusion paragraph.

“The findings suggest a tendency towards stronger grip strength being associated with larger anterior and medial thigh muscles in the male group, whereas no significant trend was observed in the female group. Specifically, in males, certain thigh muscles such as the Rectus femoris, Vastus lateralis, Vastus intermedius, Adductor group, and Gluteus maximus correlated with sarcopenia, while females did not exhibit reasonable correlations. While grip strength may have limited utility as a screening tool for sarcopenia in females, the small sample size in this study restricts the generalizability of these findings. Future research should aim to validate these observations in larger and more diverse cohorts with balanced gender representation to enhance the interpretability and applicability of the results.”

18. ***In the "Discussion" section, the authors did not provide any explanation for why no correlation was observed between individual thigh muscles and sarcopenia diagnosis (based on grip strength alone) for the female group. The authors should discuss the potential reasons for why a significant correlation was observed in the male group but not the female group, and the potential implications of this finding.***

**Response:**

Thank you for your insights. To avoid any confusion, I have made it clear that this is about the correlation between thigh muscle volumes and sarcopenia, according to the current consensus. Thank you for pointing this out.

“The present study employed grip strength and skeletal muscle index as a consensus marker for diagnosing sarcopenia and found significant correlations between sarcopenia and thigh muscle volumes such as Rectus femoris, Vastus lateralis and Vastus intermedius, particularly in the male cohort. Those specific thigh muscles imply their potential contribution in the onset or progression of sarcopenia in males. Conversely, no significant associations were observed between sarcopenia and each individual muscle volume in female group.”
