# Supplementary material for: Correlation between individual thigh muscle volume and grip strength in relation to sarcopenia with automated muscle segmentation
Source: PLoS One. 2024 Dec 26;19(12):e0312107. doi: 10.1371/journal.pone.0312107 (PMC11670947; doi:10.1371/journal.pone.0312107)
Supplement: Attachment — Submitted filename: [Response] RebuttalLetter_240905.docx [file pone.0312107.s003.docx]

**Response:**

***Thank you for submitting your manuscript to PLOS ONE. After careful consideration, we feel that it has merit but does not fully meet PLOS ONE’s publication criteria as it currently stands. Therefore, we invite you to submit a revised version of the manuscript that addresses the points raised during the review process.***

***There is really only one thing that has been requested of one Reviewer and this can be done very quickly.***

***Please identify your code and details of the software environment for your project to the readers of your manuscript. This is consistent with the Journal's goals of data reproducibility (see below). This small revision will increase your data so as to improve reproducibility etc. After receiving this in your revision, i am prepared to accept the manuscript without additional review.***

**Response:**

Thank you for your valuable feedback. I have specified the software environment we used in the ‘Methods’ section on line 216. Furthermore, the training codes of muscle segmentation model is not publicly available, as the trained model weights and the datasets utilized are not accessible through open access.

“The muscle segmentation model was trained using an Nvidia DGX A100 system, powered by two Nivia A100 Gpus and running on Ubuntu 20.04 operating system. The model was implemented with the Pytorch and MONAI libraries. For the training process, the dice coefficient was utilized as the loss function. The AdamW optimizer was set with a learning rate of 8e-5, weight decay of 1e-5, a batch size of 4 and 3000 epochs. These values were selected through a grid search method, testing different learning rates range from 1e-3 and to 1e-5”

Additionally, the affiliations of the first author, Hyeon Su Kim, and the third author, Hyunbin Kim, have been updated to 'Department of Orthopaedic Surgery, Inha University Hospital, Inha University College of Medicine, Incheon, South Korea.' We have made the changes to reflect this on the manuscript. Thank you for your understanding.
